# Acute Effect of Exercise with and without Cooperative Activities on Emotion Recognition in Preadolescent Children: A Randomized Controlled Trial

**DOI:** 10.1249/MSS.0000000000003878

**Published:** 2025-10-21

**Authors:** MELANIE BERGER, TORU ISHIHARA, KEITA KAMIJO, RAINER GREIFENEDER, MARKUS GERBER, SEBASTIAN LUDYGA

**Affiliations:** 1 Department of Sports, Exercise and Health, University of Basel, Basel, SWITZERLAND; 2 Graduate School of Human Development and Environment, Kobe University, Nada-ku, JAPAN; 3 Faculty of Liberal Arts and Sciences, Chukyo University, Nagoya, JAPAN; 4 Faculty of Psychology, University of Basel, Basel, SWITZERLAND

**Keywords:** COGNITION, EXERCISE, PREADOLESCENCE, SCHOOL, SOCIAL BEHAVIOR

## Abstract

**Purpose:**

This study aimed to investigate the acute effect of exercise, with and without cooperative activities, on emotion recognition in preadolescent children and its association with parasympathetic activity as well as prosocial behavior and inclusive thinking.

**Methods:**

Using an experimental design, 100 participants (*N* = 44 male; age = 11.6 ± 0.6 y) were randomly assigned to a group performing 20-min exercise demanding cooperation (EX+CO), aerobic exercise without cooperation (EX), or a control group (CON) watching a video in a 1:1:1 ratio. Before and after the exercise bout or control condition, a computerized emotion recognition task was administered with simultaneous recording of heart rate variability via electrocardiography, reflecting parasympathetic activity. Additionally, prosocial behavior was measured by willingness to help an excluded classmate and inclusive thinking by a social grouping task.

**Results:**

Analysis of covariance revealed a statistically significant group effect for emotion recognition accuracy (*P* < 0.05, η²_p_ = 0.07), which indicated that EX had a greater posttest accuracy compared to EX+CO and CON, when adjusted for pretest scores, age, and sex. Better behavioral performance at posttest was correlated with less inclusive thinking (*r*(73) = 0.20, *P* = 0.091), whereas no correlation with was found with parasympathetic activity during the emotion recognition task.

**Conclusions:**

A short exercise session can temporarily enhance emotion recognition abilities, which are related to social behaviors essential for classroom dynamics. The exercise-induced benefit does not seem to be related to a parasympathetic withdrawal, but depends on the required level of cooperation.

Emotion recognition allows the identification and interpretation of other individuals’ emotional states, thereby facilitating the prediction of their behavior (1). Accurate emotion perception is a critical component for navigating social interactions as it underpins more complex social-cognitive abilities, including emotion regulation and theory of mind (2). Emotion perception develops progressively during childhood from simple valence distinctions (pleasant–unpleasant) to more differentiated emotions (3). The ability to recognize emotions accurately has been related to higher academic performance, prosocial behavior, positive peer relationships, and favorable social-adaptive behavior in the classroom (3,4). For example, accurately recognizing distress cues in others can activate empathic processes, which subsequently increase the likelihood of sharing, helping, and cooperating (5). Engaging in prosocial behaviors, such as helping a socially excluded classmate, has been shown to elicit increased brain activity in regions involved in social-cognitive processing (6). Consequently, emotion recognition and prosocial behavior both contribute to the development of inclusive educational environments (7). In contrast, deficits in emotion recognition ability are linked to problematic behaviors (8) and neurodevelopmental disorders (9). Especially the transition from childhood to adolescence represents a sensitive period for social processing, involving a shift from parental and familial relationships toward increased focus on peer acceptance (10). However, evidence on interventions that may elicit immediate benefits for social-cognitive abilities and social interaction in the classroom is limited.

A theoretical model (11) suggests that exercise can influence social-cognitive abilities by acting on features of the nervous system that evolved to cope with increasing social complexity of our society: social-cognitive abilities and executive functions, which are higher-order cognitive functions essential for regulating behavior, share neural substrates and cognitive processes (12,13). The top–down regulation of behavior is essential in social contexts, for example, during challenging social conversations, as it supports deliberate actions, thoughtful communication, and adherence to social norms. Current evidence suggests that single exercise sessions can temporarily enhance executive functions (14), but effects on related social-cognitive abilities have rarely been examined. One of the few existing studies suggests that a single bout on an ergometer bicycle at moderate-to-high intensity can enhance emotion recognition in healthy adults (15). Another study, involving children with a mean age of 13 yr, investigated the effect of 35 min of aerobic exercise with and without coordinative demands on emotion recognition. The results indicated that combining aerobic exercise with coordinative tasks appears to yield greater benefits for emotion recognition, potentially due to increased neural activity within the prefrontal cortex (16).

According to Polyvagal Theory, the autonomic nervous system, which regulates physiological states in response to changing internal and external demands, has evolved a social engagement system. This system relies on the myelinated fibers of the vagus nerve, a key component of the parasympathetic nervous system, and is activated during autonomic balance. The Polyvagal Theory posits a bidirectional communication pathway between the heart and the brain (17). Heart rate variability (HRV) measures temporal fluctuations between consecutive heartbeats and can be used as an index of the autonomic nervous system (18). Acute aerobic exercise leads to a transient reduction of HRV (19). Parasympathetic withdrawal, as indicated by reduced HRV, shifts autonomic balance toward sympathetic dominance, promoting survival-related behaviors (e.g., fight-or-flight), while suppressing the social engagement system. More specifically, reduced HRV might affect emotion recognition, as vagal afferents converge in the brainstem and modulate the activity of higher brain structures. In particular, projections from the nucleus tractus soliatrius to forebrain regions, including limbic and prefrontal areas, provide a neurophysiological basis for the integration of visceral information into cognitive and emotional processing (17). Therefore, a single exercise session could lead to a downregulation of the social engagement system, potentially impairing emotion recognition, until HRV is recovered. This assumption contradicts the available evidence, which, however, did not include HRV measurements. Since no study has directly measured HRV in this context to date, it remains necessary to investigate how exercise-induced changes in HRV are actually related to changes in emotion recognition.

While a decrease in HRV following exercise would typically be associated with impaired emotion recognition, alternative mechanisms could potentially explain the observed improvements in emotion recognition. Nonexercise interventions designed to foster social cognition mainly focus on social skill training and emotion regulation (20). Social interaction both relies on and provides an opportunity to apply social-cognitive abilities (21). Cooperation, as a specific form of social interaction, involves coordinated group activities designed to achieve a shared goal that would be difficult to accomplish without the support of others. This approach can be incorporated into exercise-based interventions.

We investigated the acute effect of exercise, with and without the integration of cooperative activities, on emotion recognition in children in a school setting. We expected greater pre- to posttest improvements in emotion recognition after cooperative compared with noncooperative exercise activities as well as greater benefits after noncooperative exercise compared with the control condition. In addition to behavioral performance differences, we investigated parasympathetic activity as underlying mechanism and transfer effects of improvements in emotion recognition to prosocial behavior and inclusive thinking. We hypothesized that greater exercise-induced parasympathetic withdrawal would be negatively associated with emotion recognition accuracy. Additionally, we predicted that improvements in emotion recognition accuracy would be positively correlated with increased prosocial behavior and inclusive thinking.

## METHODS

### Participants

Recruitment was carried out by contacting school managements in northwestern Switzerland and then holding of information events with the legal guardians. The eligibility criteria of the participants included age between >9 and ≤13 yr and corrected-to or normal vision. Participants were excluded if they were unable to engage in physical exercise, had an acute or chronic condition posing a safety risk during exercise, or were undergoing pharmacotherapy, behavioral therapy, and/or social skills training for a mental disorder (as reported by parents). For the present analysis, the sample was restricted to children with a sum score under 16 in the Strength and Difficulties Questionnaire (SDQ) to reduce the risk of participants with an abnormal level of emotional and behavioral difficulties. Legal guardians and participants were fully informed about the study in an information event, and written informed consent was obtained. The study protocol was approved by the local ethics committee and complied with the Helsinki Declaration and its amendments.

**FIGURE 1:**
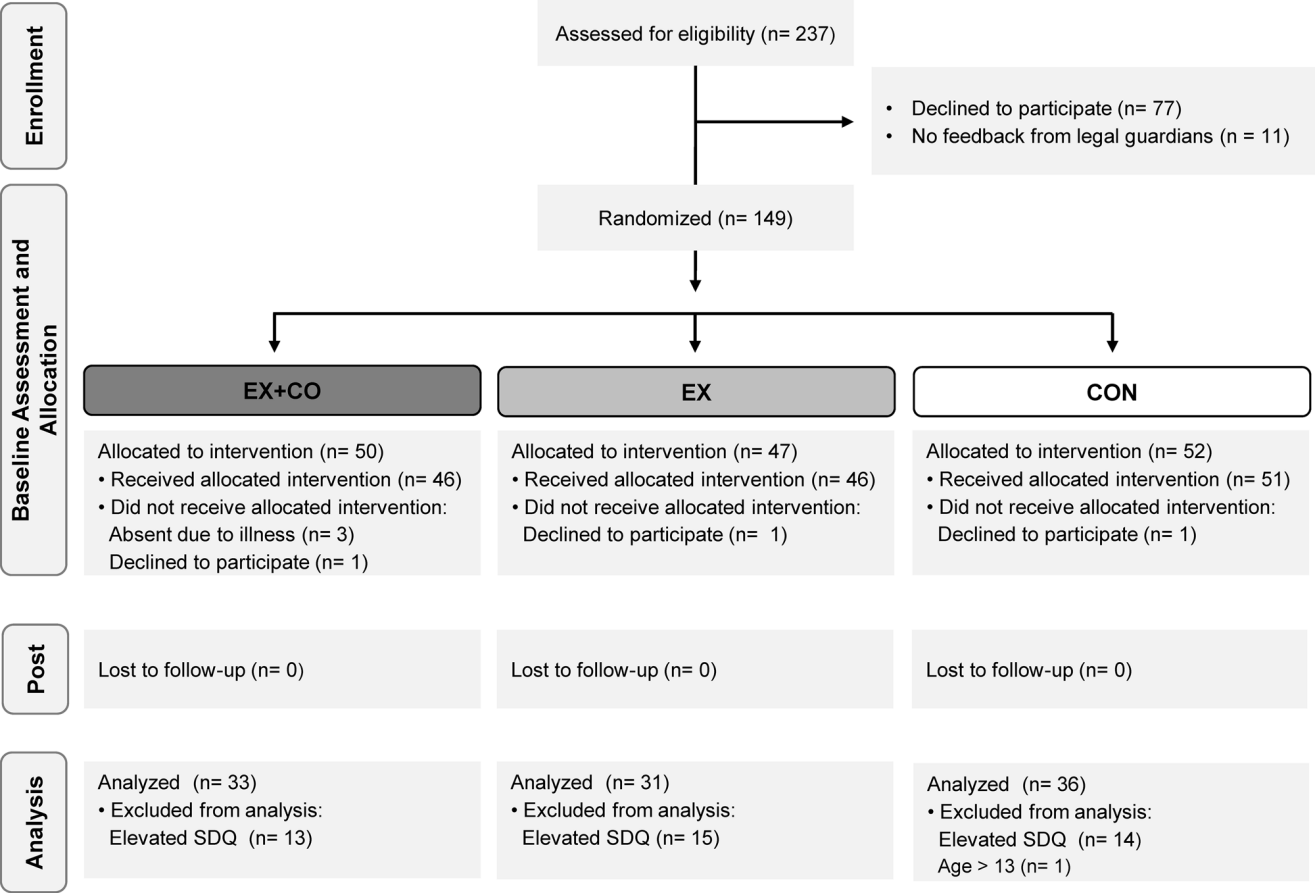
Participant flow chart. EX+CO, exercise with cooperation; EX, aerobic exercise without cooperation; CON, control group.

An a priori sample size calculation was conducted with G*Power. The two existing experimental studies suggest a moderate effect of exercise on emotion recognition (15,16). The alpha error probability was set to α = 0.017, accounting for the inclusion of four covariates. To achieve 80 % power on an analysis of covariance (ANCOVA) controlling for pretest scores, 29 participants per group (87 in total) were required (Fig. 1).

### Study Design

In this RCT with pre- and posttest designs, participants were randomly allocated (stratified by school class) to a group performing exercise, which required cooperation among the participants (EX+CO), a group performing aerobic exercise without cooperation (EX), and a control group (CON) in a 1:1:1 ratio (Supplemental Appendix 1, Supplemental Digital Content, https://links.lww.com/MSS/D313). The randomization list was created with a sealed envelope and used permuted blocks. Although participants were not blinded, the individuals responsible for processing the cognitive data were blinded to group allocation.

At both the baseline assessment and posttest (5 min after exercise cessation), participants completed a facial emotion recognition task during which HRV was recorded. Following this, prosocial behavior and inclusive thinking were assessed. Additionally, participants completed a 7-d physical activity recall and answered the Family Affluence Scale. Anthropometric data, including height and weight, were also measured. The testing sequence was identical for all participants.

### Intervention

Participants were divided into three groups. The intervention lasted 20 min. Both exercise interventions EX+CO and EX were designed to be age-appropriate and playful. A description of the games can be found in the supplemental appendix (Supplemental Appendix 2, Supplemental Digital Content, https://links.lww.com/MSS/D313). The control group watched a sports documentary with minimal affective stimuli.

### Measures

#### Facial emotion recognition

In the computer-based facial emotion recognition task administered with E-Prime 3.0.3.214 (PST, Pittsburg, PA), children had to identify emotions from faces and press a button corresponding to anger, fear, or happiness. Accuracy and speed of responses were equally emphasized during the instructions. The visual stimuli were obtained from the FACES database and contained equal proportions of Caucasian male and female faces as well as faces from young, middle-aged, and older adults. The order of the emotions was randomized, and visual stimuli were presented against black background. Each session commenced with three example stimuli, followed by 15 practice trials. Participants then completed two blocks with 39 trials each. Visual stimuli remained on screen for 500 ms, with responses collected within a 1500 ms time window. The intertrial interval varied randomly between 900 and 1300 ms to reduce the risk of guessing. Accuracy averaged across the three emotions was calculated as the main outcome, whereas averaged reaction time on response-correct trials served to control for an accuracy-speed trade-off.

#### Task-related heart rate variability

The ECG was recorded at 130 hz using a heart rate monitor with a flexible chest belt (H10 sensors, Polar Electro Oy, Kempele, Finland), because this rate is considered suitable for obtaining reliable results for time-domain parameters (22,23). Processing was performed with Kubios HRV Standard 3.5.0. Artifacts were defined as interbeat intervals deviating by more than 0.15 s from the local mean and identified via a threshold-based algorithm. Corrections were applied using cubic spline interpolation. If over 5% of intervals required adjustment, the threshold was increased to 0.25 s. The natural logarithm of the root mean square of successive R-wave to R-wave interval differences (LnRMSSD) were calculated to estimate the vagally mediated changes in HRV. Lower LnRMSSD indicates greater vagally mediated parasympathetic withdrawal. RMSSD is relatively free of respiratory influences and is sensitive to parasympathetic reactivation after exercise (23,24). LnRMSSD provides good reliability even in shorter recording durations compared to the traditional 5-min RMSSD recording recommendation (23,25).

#### Transfer effects

Prosocial behavior was assessed by the tendency to help an excluded classmate, on a scale from 0 (under no circumstances) to 10 (definitely). Higher scores indicate greater prosocial behavior. Furthermore, to measure inclusive thinking, the children were asked to categorize their classmates into groups, using a list of their names. The number of groups was adjusted for class size. Children reporting fewer groups are considered to display a more inclusive mindset.

### Statistical Analysis

All statistical analyses were performed with SPSS 29.0.2.0 (IBM, Pittsburgh, PA). In preliminary visual inspection, there was no indication for nonlinear relations between emotion recognition and other variables of interest. To test whether the exercise manipulation was successful, an analysis of variance examined group differences in heart rate. The main analyses employed ANCOVAs on posttest scores of emotion recognition (accuracy and reaction time separately), LnRMSSD, prosocial behavior, and inclusive thinking. Each ANCOVA included group as between-subjects factor and controlled for pretest scores, age, and sex. The covariates were chosen because of evidence suggesting that age influences the exercise-induced adaptations (26) and that sex influences facial expression processing (27). Marginal means were estimated for preplanned comparisons of EX versus CON and EX-CO versus EX. Finally, partial correlation coefficients, controlling for age and sex, were calculated for linear associations between emotion recognition accuracy, LnRMSSD, prosocial behavior, and social grouping at posttest. Referring to social grouping, the partial correlation was further adjusted for class size. The level of significance was set to *P* <0.05.

## RESULTS

### Participants’ Characteristics

Of 143 participants, one child was excluded from data analysis due to being older than 14 yr. Forty-two participants were excluded because they reported SDQ scores indicating an abnormal range of emotional and behavioral difficulties. The baseline characteristics of the remaining 100 participants are displayed in Table 1. The exercise manipulation was successful as indicated by a significantly higher heart rate in the EX+CO and EX groups compared with CON (*F*_(2, 97)_ = 189.38, *P* < 0.001, η²_p_ = 0.80).

**TABLE 1.** Baseline sample characteristics divided by groups.

	EX+CO *n* = 33		EX *n* = 31		CON *n* = 36	
	M	SD	M	SD	M	SD
Age (yr)	11.5	0.6	11.6	0.6	11.6	0.6
Male sex (n, %)	18	54.5%	14	45.2%	12	33.3%
Height (cm)	151.2	6.3	155.3	9.6	152.3	7.7
Weight (kg)	43.2	10.4	46.9	10.9	44.7	10.9
BMI (kg/m^2^)	18.8	3.7	19.3	3.4	19.1	4.0
SDQ	10.4	2.8	9.6	4.0	9.7	3.2
FAS	6.4	2.0	7.0	1.5	7.0	1.8
MVPA (min/d)	42	31	57	41	49	34
HR during intervention (bpm)	140	17	143	17	84	8

BMI, body mass index; CON, Control group; EX, Aerobic exercise without cooperation; EX+CO, Exercise with cooperation; FAS, Family Affluence Scale; MVPA, moderate-to-vigorous physical activity; RT, Reaction time; SDQ, Strengths and Difficulties Questionnaire; T0, pre; T1, post.

### Emotion Recognition

**FIGURE 2:**
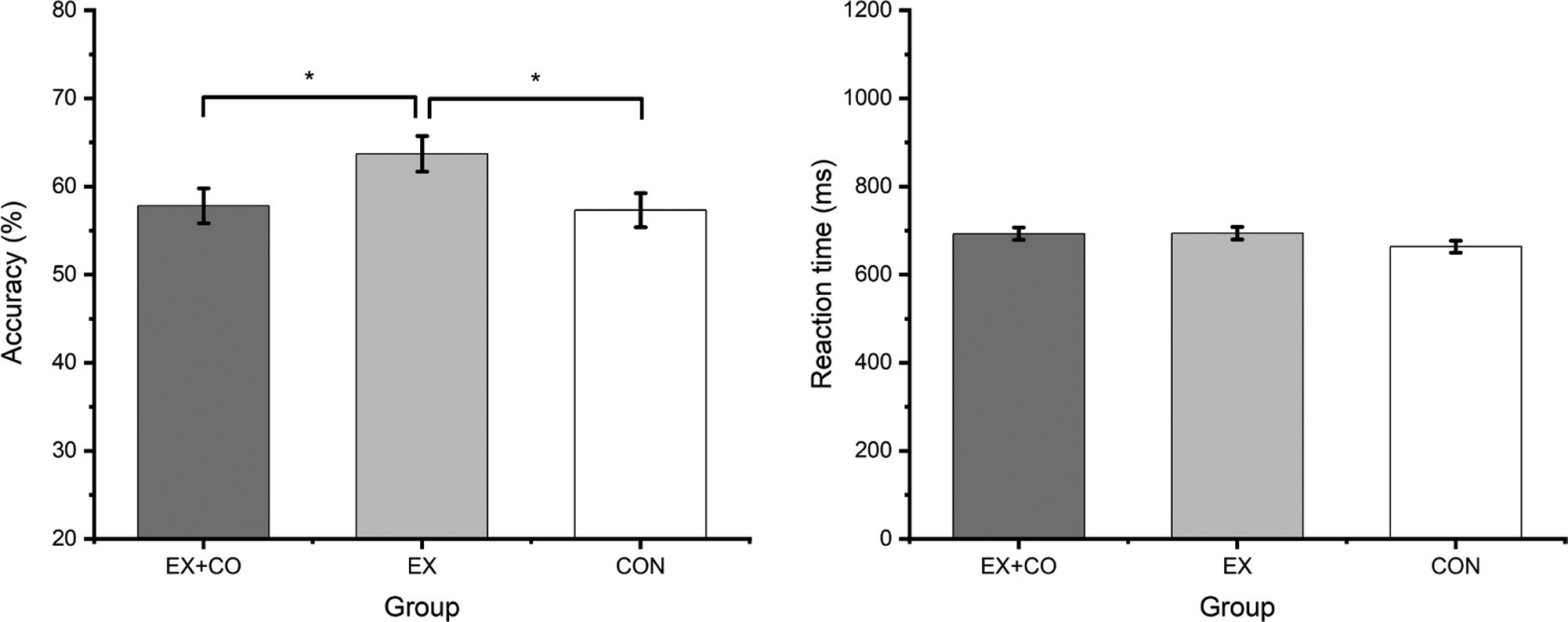
Marginal means and standard errors for emotion recognition accuracy and reaction time divided by groups and adjusted for age, sex, and baseline performance. *Significant on ***P*** <0.05.

The ANCOVA revealed a significant group effect for posttest emotion recognition accuracy, while controlling for baseline scores, age, and sex, *F*_(2, 92)_ = 3.25, *P* < 0.05, η²_p_ = 0.07. Preplanned comparisons showed that EX had a significantly greater posttest accuracy compared to CON (∆M = 0.06, ∆SE = 0.03, *P* = 0.024) and EX+CO (∆M = 0.06, ∆SE = 0.03, *P* = 0.038) in the adjusted analyses. In contrast, there was no main effect of group on posttest reaction time, *F*_(2, 92)_ = 1.62, *P* > 0.05, η²_p_ = 0.03, ruling out accuracy-speed trade-offs as alternative explanation (Fig. 2).

### HRV

**FIGURE 3:**
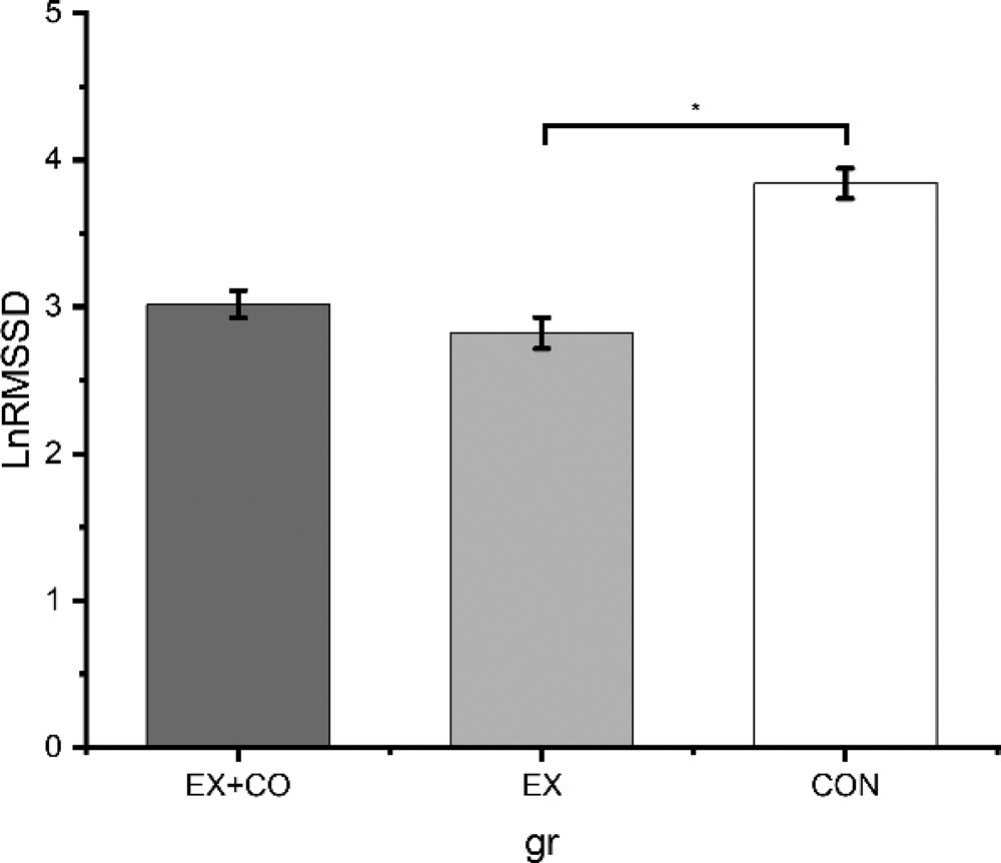
Marginal means and standard errors for LnRMSSD divided by groups and adjusted for age, sex, and baseline performance. LnRMSSD, natural logarithm of root mean square of successive differences. *Significant on ***P*** <0.05.

The analysis revealed a statistically significant main effect of group, *F*_(2, 68)_ = 27.41, *P* = 0.01, η²_p_ = 0.45. Comparisons showed that EX had a significantly lower LnRMSSD than CON (∆M = −1.02, ∆SE = 0.15, *P* ≤ 0.001). No credible difference was found between EX and EX+CO (∆M = −0.20, ∆SE = 0.14, *P* = 0.179) (Fig. 3).

### Prosocial Behavior and Social Grouping

**FIGURE 4:**
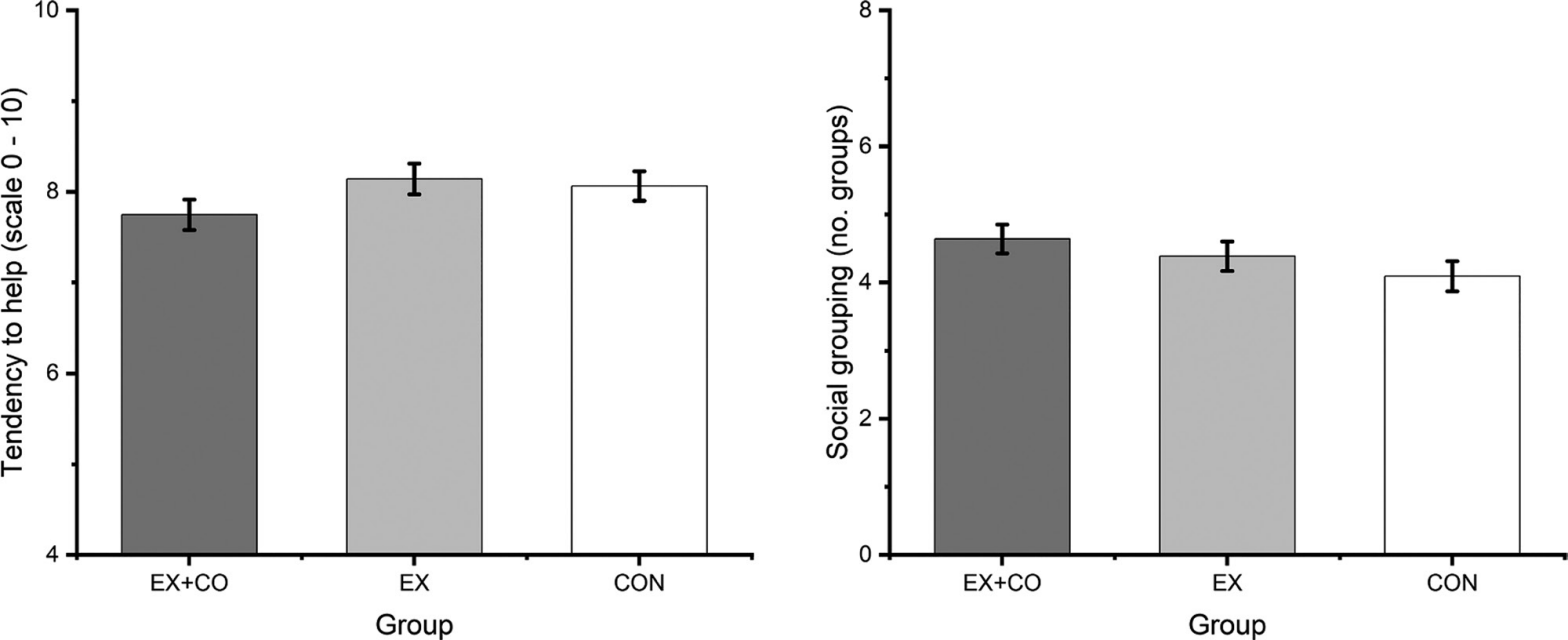
Marginal means and standard errors for prosocial behavior and social grouping divided by groups and adjusted for age, sex, and baseline performance. The unadjusted means and standard deviations from emotion recognition ability, HRV indices, prosocial behavior and social grouping at baseline and posttest can be found in Supplemental Appendix 3, Supplemental Digital Content, https://links.lww.com/MSS/D313.

There were no significant group differences for self-reported tendency to act prosocially, *F*_(2, 93)_ = 1.52, *P* > 0.05, η²_p_ = 0.03, and social grouping at posttest, *F*_(2, 67)_ = 1.55, *P* > 0.05, η²_p_ = 0.04, while controlling for age, sex, and baseline scores (Fig 4).

### Correlations between Posttest Emotion Recognition, HRV, and Transfer Effects

As shown in Table 2, emotion recognition accuracy was not correlated with LnRMSSD. Furthermore, no association was found between emotion recognition and prosocial behavior. However, emotion recognition accuracy did appear to have a positive linear association with social grouping (*r*_(73)_ = 0.20, *P* = 0.091), indicating that children who performed more accurately on the emotion recognition task tended to divide their class into a greater number of groups, reflecting less inclusive thinking as defined in the study.

**TABLE 2.** Partial correlation coefficients (*r*) between posttest emotion recognition accuracy and LnRMSSD, prosocial behavior as well as social grouping, controlled for age and sex and for class size in context of social grouping.

ER ACC	LnRMSSD	Prosocial Behavior	Social Grouping
*r*	−0.139	0.145	0.197
*P*	0.241	0.155	0.091
*df*	71	96	73

ACC, accuracy; ER, emotion recognition; LnRMSSD, natural logarithm of root mean square of successive differences.

## DISCUSSION

After controlling for baseline scores, EX showed better emotion recognition accuracy at posttest compared to both EX+CO and CON, with no indications provided for a speed-accuracy trade-off. Moreover, EX exhibited significantly higher LF power and significantly lower HF power than CON, indicating an exercise-induced parasympathetic withdrawal. However, the physiological state of autonomic nervous system was not correlated with the behavioral performance in emotion recognition accuracy. Additionally, there were no significant differences between the groups regarding self-reported prosocial behavior and social grouping at posttest. Nonetheless, higher emotion recognition accuracy tended to be associated with greater number of social groups perceived within the class. On a speculative note, this may indicate that higher emotion recognition accuracy is associated with perceiving more within-group differences.

Our results contribute to the evidence of an acute beneficial effect of exercise on emotion recognition (15,16). However, this effect appears not be augmented by the level of cooperation, as contrary to our assumptions, the EX+CO condition did not improve emotion recognition. A possible explanation is that the EX+CO condition may have depleted cognitive resources. The cooperative activities were intentionally designed to be unsolvable alone, thus requiring social coordination. Social coordination is a key characteristic of social interactions, as it reflects the extent to which individuals can efficiently and effortlessly synchronize their behavior (28). When the coordination process is inefficient, social interaction can become more cognitively demanding and energy-draining than individual performance. Following such inefficient interactions, individuals tend to avoid challenging tasks or approach them with reduced focus and concentration (28). However, the efficiency of cooperation was not assessed in this study. Additionally, the children in the EX+CO condition were compelled to cooperate. For those who prefer independent task completion, this necessitated exerting self-control to fulfill the instructed game format. Self-control refers to the process of regulating one’s thoughts, feelings, or behaviors to alter them from their natural tendencies. It is regarded as a limited cognitive resource that can become depleted, resulting in diminished capacity in subsequent situations (29). Consequently, performance on the emotion recognition task of EX+CO may have been compromised due to the depletion of cognitive resources caused by self-control exertion. Furthermore, due to random group assignments, the children may have had to interact with peers they did not like. Depending on the situation, the affective state likely differed, influencing cognitive processing (30). Based on cognitive load theories, both positive and negative mood states consume cognitive resources, impairing cognitive performance, as they redirect attention toward mood-related thoughts rather than task-relevant ones (31). In contrast, further evidence suggests a connection between negative mood and enhanced accuracy in face recognition, proposing that individuals in a sad mood process information more thoroughly than those in a happy state (32). Additionally, according to the theory of emotion-specific bias, both sad and happy moods selectively impair the recognition of emotions that are incongruent with the individual’s current affective state, while the identification of mood-congruent expressions appears to remain unaffected (33). However, the circumstances described reflect the real-world scenario in a school class in which individuals must interact with others with whom cooperation is more challenging and, in some cases, more or less enjoyable. Future research should investigate whether social interactions become more efficient over time, when performed regularly, and whether the beneficial effects of exercise are no longer canceled out or even enhanced by this social skills training.

With respect to HRV, our findings contradict the assumption derived from Polyvagal Theory that parasympathetic withdrawal might temporarily impair emotion recognition. Previous studies supporting an association between autonomic balance and emotion recognition within the framework of Polyvagal Theory focused on resting-state HRV (34,35). In addition, transcutaneous vagus nerve stimulation has been shown to enhance emotion processing, further supporting the hypothesis that the vagus nerve plays a causal role in emotion recognition (36,37). The present study tested this theory by investigating task-related HRV manipulated by exercise. The EX group showed improved emotion recognition accuracy despite experiencing parasympathetic withdrawal. However, it is important to note that no significant correlation was observed between HRV and behavioral performance in this study. Therefore, the underlying mechanisms driving the positive effects of exercise on emotion recognition warrant further investigation. Future studies might consider other mechanisms, as modulation of the central nervous system or an increase in oxytocin (11).

The interventions showed no significant effect on the self-reported prosocial behavior or inclusive thinking, after controlling for baseline scores. The potential requirement to cooperate with disliked peers due to random group assignment may have contribute to the by tendency lower intention of prosocial behavior in EX+CO. Furthermore, higher emotion recognition ability at posttest was not significantly associated with increased self-reported tendency for prosocial behavior. This finding contrasts with previous evidence indicating a positive association between accurate emotion recognition, especially the emotion fear, and prosocial behavior (5,38). However, higher emotion recognition accuracy seems to be positively corelated with the number of social groups perceived within the class. The social grouping task, designed to test inclusive thinking, was initially expected to reveal a negative association. The present finding of a positive association could indicate that individuals with higher emotion recognition skills may be more sensitive to perceive nuanced distinctions between groups.

### Limitations

A profound strength of this study is that it was conducted in a real-world setting, thus providing insights into practical implications. However, several limitations should be considered when interpreting our findings. First, the between-subjects study design has the limitation that individual differences, personality traits, and unaccounted-for covariates may have influenced the results. Nevertheless, it allowed the assessment of the general applicability in a typical school class, while minimizing the risks of expectation and learning effects, as well as reducing participant burden, in comparison to a within-subjects design. Second, the exclusion of children with behavioral therapy, pharmacotherapy, and social skill training as well as children with an elevated SDQ score may have reduced the generalizability, since about one in three children seemed to be affected. Therefore, the acute effect of exercise on emotion recognition in children with neurodevelopmental diseases should be investigated further. This is particularly relevant as children with lower baseline cognitive abilities may experience greater benefits from a single exercise session (39). Third, the facial emotion recognition task used images of adult faces, which may have introduced an own-age bias, as participants tend to recognize emotions more accurately in faces of individuals their own age. Although the accurate interpretation of emotions displayed by adults is important, for example, when interacting with teachers, images of same-aged children would have been more appropriate since the intervention was conducted with peers. Fourth, the instruments used to assess prosocial behavior and inclusive thinking have not been formally validated. Although validated tools are available, they predominantly target stable traits and demonstrate internal validity, with limited applicability to dynamic, state-like constructs. In contrast, current recommendations for assessing changeable states emphasize the use of simple, context-sensitive methods that incorporate information from children’s social environments to enhance ecological and external validity. Our methodological approach aligns with these recommendations, despite the lack of formal validation. Fifth, the cooperation did not arise naturally, but was specifically encouraged by the exercise activities. Thus, the cooperation primarily served to achieve the exercise-specific goals. Sixth, the children`s affective state should have been assessed. However, regarding the perception of social coordination efficiency, the subjective evaluation of the social coordination process appeared to have no impact on subsequent self-regulatory processes. If a child perceives the coordination process as stressful or experiences dislike toward partners, such perceptions do not impair self-regulatory capacity, provided that the interaction was not objectively demanding (28). Seventh, the acute effect was assessed within 5 min following the exercise session, limiting our ability to draw conclusions about the duration of the effect. Eighth, prepubertal children demonstrate rapid parasympathetic reactivation within the first minutes following exercise (40). In this context, we cannot rule out that the children’s HRV was already recovering by the time the posttest emotion recognition task was initiated. However, as shown in Figure 3, the posttest task-related HRV in both exercise groups was significantly lower than in the control group, indicating that full autonomic recovery had not occurred. Moreover, the potential for recovery was minimized by instructing the participants to return to the post assessment immediately following the exercise intervention. Ninth, time of day influences both cognitive task performance and HRV (41,42). To minimize the impact of diurnal variations, all measurements were conducted during morning hours, and the randomization was performed within classes.

## CONCLUSIONS

A single 20-min exercise session, conducted without cooperative activities, leads to temporary enhancements in emotion recognition accuracy in preadolescent children within a school setting. In contrast to the Polyvagal theory, no association between task-related autonomic state and behavioral performance in emotion recognition was observed.

The study was funded by the Swiss National Science Foundation (32513B_219263). No conflicts of interest were disclosed. The results of the study are presented clearly, honestly, and without fabrication, falsification, or inappropriate data manipulation. The results of the present study do not constitute endorsement by the American College of Sports Medicine.

## Supplementary Material
