# Supplementary material for: Acute Effect of Exercise with and without Cooperative Activities on Emotion Recognition in Preadolescent Children: A Randomized Controlled Trial
Source: Med Sci Sports Exerc. 2025 Oct 21;58(3):464–72. doi: 10.1249/MSS.0000000000003878 (PMC12863594; doi:10.1249/MSS.0000000000003878)
Supplement: Supplementary file 1 [file msse-58-464-s001.pdf]

Appendix 1 – Study Design

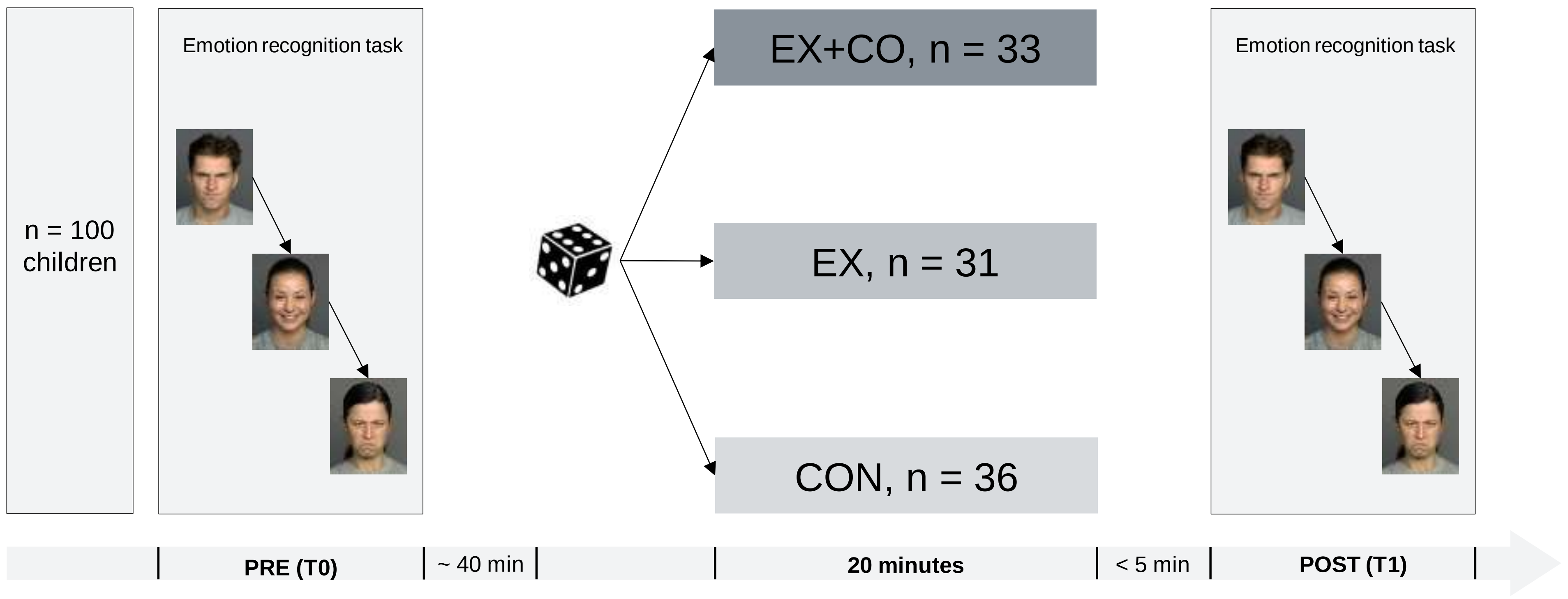

Appendix 2 – Description of the interventions

Game	Approx. duration	EX+CO	EX
Music- stopping challenge	~ 8 min	All players run through the gym. When the music stops, the players have to get together as quickly as possible. The game leader then gives the group a task. Possible tasks include:<br>- The team has to build a certain letter, like an A<br>- The team must touch the floor with exactly 4 feet and 4 hands	All players run through the gym. When the music stops, the game leader calls out a task into the room. Now the players have to solve this task as quickly as possible. Possible tasks include:<br>- Children have to stand on one leg without falling over<br>- The children have to look for an object in a certain color
Meier family	~ 6 min	Each participant receives a piece of paper with the name of a family member* (Meier, Mayer, Maier, Meyer, ...), divided into grandpa, grandma, dad, mom, son and daughter. Each participant may only read their own piece of paper. After the starting signal, the family members must come together. The family that sits down first at the correct house wins.<br><br>**In German-speaking countries, surnames like "Meier," "Mayer," "Maier," and "Meyer" are quite common. Although these names are spelled differently, they are typically pronounced the same.*	Each participant receives a piece of paper with the name of a family member (Grandpa Meier, Grandpa Mayer, Grandpa Maier, Grandpa Meyer, ...). Each participant may only read his or her own piece of paper. Additional pieces of paper with family members, divided into grandma, dad, mom, son and daughter, are distributed face down in the hall. After the starting signal, the search for the pieces of paper with the other family members begins. The players are not allowed to help each other or exchange information. The player who has all the pieces of paper together first sits down next to the house with his or her family name and wins.
Marble transport	~ 6 min	Using cardboard tubes, the group should transport ten marbles over a distance of at least 20m from point A to point B. The following rules apply to transporting marbles: 1) The marbles must not be touched with hands or other body parts. 2) The children can only move further with their cardboard tubes if there are no marbles in their tube. 3) A marble that falls down means that the marble transport process has to start again from the starting point.	Using cardboard tubes, each player must transport three marbles over a distance of at least 20m from point A to point B. The following rules apply to transporting marbles: 1) The marbles must not be touched with hands or other body parts. 2) The children must not hold the open ends of the cardboard tubes. 3) A marble that falls down means that the marble transport must begin again from the starting point.

Appendix 3 – Unadjusted means and standard deviations of emotion recognition, heart rate variability, prosocial behavior and social grouping divided by groups at T0 and T1

	**EX+CO**		**EX**		**CON**	
	T0	T1	T0	T1	T0	T1
	M<br>± SD	M<br>± SD	M<br>± SD	M<br>± SD	M<br>± SD	M<br>± SD
Ø ACC, %	56.7	58.5	57.9	65.1	54.3	56.2
	15.1	13.8	13.7	14.6	15.5	16.9
ACC happy, %	70.6	71.0	72.1	78.6	67.4	67.4
	22.1	16.7	16.9	14.6	18.0	20.4
ACC fear, %	46.8	50.9	49.8	56.4	46.2	48.6
	16.7	17.2	16.9	22.4	18.9	19.5
ACC anger, %	52.8	53.6	52.0	60.7	49.3	52.5
	15.9	21.1	16.4	17.6	19.0	18.1
Ø RT, ms	700.1	686.3	720.9	701.5	702.2	661.2
	59.3	82.6	73.5	55.9	59.2	104.2
RT happy, %	687.7	657.8	708.1	679.6	689.7	656.9
	63.3	78.9	72.9	69.8	59.3	97.5
RT fear, %	715.1	705.1	727.0	722.3	714.9	683.9
	82.1	94.5	90.1	70.3	77.5	115.5
RT anger, %	697.4	695.9	727.7	702.8	702.2	642.9
	74.9	97.6	83.1	62.6	85.4	122.0
LF/HF Ratio	1.9	3.7	2.1	3.8	1.6	1.9
	1.6	3.8	1.2	2.5	0.9	1.1
LF, %	53.7	61.5	58.1	69.4	52.3	56.1
	14.1	15.9	11.0	9.7	13.8	11.8
HF, %	38.9	29.9	34.6	23.1	40.4	36.8
	15.5	18.5	12.3	8.9	13.7	13.5
Prosocial behavior	8.4	8.2	8.2	8.3	7.4	7.6
	1.7	1.9	2.1	2.1	2.5	2.8
Social Grouping	4.3	4.5	5.0	3.9	4.6	4.2
	1.4	1.8	4.5	2.0	1.7	1.8

Notes: EX+CO = Exercise with cooperation; EX = Exercise; CON = Control group; ACC = Accuracy; RT = Reaction time; LF/HF = low frequency / high frequency ratio; LF = low frequency; HF = high frequency; T0 = pre; T1 = post.
